# Fibulin 1 and 2 Levels in Patients with Heart Failure: Comparison of Different Heart Failure Stages and Exploring the Temporal Changes During Acute Exacerbation

**DOI:** 10.31083/RCM26364

**Published:** 2025-02-21

**Authors:** Burcu Cihan Talay, Emrullah Kızıltunç, Canan Yılmaz, Zakir Osmanov, Serkan Ünlü, Mustafa Candemir, Burak Sezenöz, Özden Seçkin Göbüt, Salih Topal, Sedat Türkoğlu

**Affiliations:** ^1^Department of Cardiology, Bilecik Bozüyük State Hospital, 11300 Bilecik, Turkey; ^2^Department of Cardiology, Gazi University School of Medicine, 06560 Ankara, Turkey; ^3^Department of Medical Biochemistry, Gazi University School of Medicine, 06560 Ankara, Turkey

**Keywords:** heart failure, fibulin-1, fibulin-2, extracellular matrix

## Abstract

**Background:**

Fibulin 1 and Fibulin 2 are members of the extracellular matrix (ECM) glycoprotein family. ECMs drive prognosis through remodeling, a key step in the pathogenesis of heart failure (HF). We aimed to compare Fibulin 1 and 2 levels in different stages of HF and to investigate their relationship with other prognostic factors of HF.

**Methods:**

Patients with HF were divided into two groups according to left ventricular ejection fraction (LVEF): reduced and non-reduced LVEF. The control and patient groups consisted of individuals with Stages A and B HF, Stages C and D HF, respectively. Fibulin levels were measured at different stages of HF and in the control group. Additionally, Fibulin levels were measured at admission, discharge, and in the first month in patients who were hospitalized due to decompensated HF.

**Results:**

Serum Fibulin 1 and N-terminal pro-B-type natriuretic peptide (NT-proBNP) levels were significantly higher in the patient group than in the control group. Serum Fibulin 2 levels were similar between the groups. Although serum Fibulin 2 levels were similar at repeated measurements, serum Fibulin 1 and NT-proBNP levels significantly decreased at discharge and remained similar at 1 month compared with admission. There was a significant positive correlation between Fibulin 1 and NT-proBNP levels and a significant negative correlation between Fibulin 1 levels and LVEF. Fibulin 2 levels were not correlated with LVEF and NT-proBNP.

**Conclusions:**

Our study demonstrated that serum Fibulin 1 levels differ among different HF stages and have a similar temporal change as observed for NT-proBNP levels. A similar association was not observed for Fibulin 2 in our study.

## 1. Introduction

Almost all heart diseases lead to myocardial fibrosis as a response to myocyte damage. This process eventually leads to heart failure (HF). The extent of myocardial fibrosis is closely associated with HF and unfavorable outcomes [1, 2]. Although different diagnostic tools exist to define myocardial fibrosis, such as cardiac magnetic resonance imaging or endomyocardial biopsy, circulating biomarkers for determining myocardial fibrosis are an active research field in clinical cardiology [3]. The extracellular matrix (ECM) is the main object of adverse cardiac remodeling, with changes in its structure and function following cardiac injury [4]. Fibulins are ECM glycoproteins present in the basement membrane and elastic fibers. Fibulin 1 and 2 are known as “long fibulins” of the eight-membered Fibulin family [5]. Previous studies have demonstrated that Fibulin 1 and 2 are associated with fibrotic processes in mammalian disease states. Liu *et al*. [6] reported increased levels of Fibulin 1 in bronchoepithelial cells and serum in patients with chronic obstructive pulmonary disease. They also showed that Fibulin 1 inhibition resulted in a decrease in collagen deposition around the small airways in an experimental obstructive pulmonary artery disease model [6]. Another experimental study implicated Fibulin 2 as a critical factor in hypertrophic response to angiotensin II in the heart [7]. Fibulin 1 and 2 are present in the circulation beyond their presence in the ECM [5]. Additionally, Ibrahim *et al*. [8] found that Fibulin 2 levels are correlated in the serum and tissue of patients with hypertrophic cardiomyopathy, suggesting that serum levels could reflect tissue expression. Myocardial fibrosis is closely linked with HF and its severity. However, the levels of Fibulin 1 and 2 in patients with HF remain unclear. Therefore, we aimed to determine serum Fibulin 1 and 2 levels in different stages of patients with HF and to investigate temporal changes in serum Fibulin 1 and 2 levels in patients hospitalized with decompensated HF.

## 2. Methods

This prospective observational study was conducted at Gazi University Cardiology Department. Informed consent was obtained from all patients. The ethics committee of Gazi University School of Medicine approved the study protocol. The Gazi University Scientific Projects Department supported this study (Project number TTU-2022-7505).

### 2.1 Study Patients

The study population comprised patients who were hospitalized due to symptomatic HF (patient group) and patients who had at least one major cardiovascular disease as a risk factor for HF development but did not have current or prior symptoms (control group). The patient group was divided into two groups according to left ventricular ejection fraction (LVEF). Patients whose LVEF was lower than 40% were included in the reduced LVEF group, and the remaining patients were included in the non-reduced LVEF group. The control group included individuals with Stage A and B HF, and the patient group included individuals with Stage C and D HF, according to the universal definition and classification of HF [9]. The following patients were excluded from the study. Patients younger than 18 years, patients diagnosed with pregnancy-associated HF, patients whose HF was primarily associated with heart valve diseases like severe aortic regurgitation/stenosis, severe primary mitral regurgitation, and severe rheumatic mitral stenosis/regurgitation, patients with active infection or a sepsis on admission, patients with active malignancy, patients with rheumatic disease, patients who were on renal replacement treatment, and patients diagnosed with concomitant myocardial infarction.

Symptoms, functional capacity, physical examination findings, cardiovascular risk factors, and medications were recorded at the initial visit. Echocardiographic evaluations of patients at rest were performed by the same cardiologist, who was blinded to the clinical data, using the General Electrics Vivid E95 device 2D M5Sc-D probe (GE Vingmed Ultrasound,GE HealthCare Technologies, Chicago, IL, USA). The modified biplane Simpson method was used to measure LVEF. Complete blood count, serum biochemistry, and N-terminal pro-B-type natriuretic peptide (NT-proBNP) levels were recorded from the local laboratory results. All the aforementioned recordings were performed at discharge and in the first month after discharge in patients hospitalized for decompensated HF. No additional visits or analysis were performed for the control group. Need for intensive care follow-up due to HF progression, need for hemodialysis during the index hospitalization, stroke, recurrent HF hospitalizations, or any mortality in the first month were defined as adverse study outcomes.

### 2.2 Fibulin Analyses

Blood samples were obtained at the first visit from all patients, pre-discharge, and the first month from individuals in the patient group. Serum samples were separated by centrifugation at 4000 rpm for 10 minutes and stored at –80 °C until processing. All Fibulin analyses were performed simultaneously by Fibulin 1 (FBLN1) and Fibulin 2 (FBLN2) Human sandwich ELISA kits (ELK Biotechnology Co., Ltd., Wuhan, China). The Fibulin 1 (Cat No: ELK3375, Lot No: 20334253757) ELISA kit sensitivity was 1.34 ng/mL; measurement range was 2.13–200 ng/mL; whilst the Fibulin 2 (Cat No: ELK 3852, Lot No: 20334254714) ELISA kit sensitivity was 0.241 ng/mL; measurement range was 0.63–40 ng/mL; the percent coefficient of variation (CV%) values ​​for both kits were given as <8% within the study and <10% between the studies. Serum NT-proBNP levels were measured by electrochemiluminescence using the Roche Cobas 6000 autoanalyzer (Roche Holding AG, Basel, Switzerland).

### 2.3 Statistical Analyses

Categorical variables were presented as numbers and percentages; continuous variables were presented as medians (interquartile range) or means ± standard deviations. The distribution pattern of continuous variables was determined using the Kolmogorov-Smirnow test. We used only non-parametric tests to analyze continuous variables, as each study group had a sample size <30. The chi-square or Fisher’s exact test was used to compare categorical variables between the groups. Mann-Whitney U test was used to compare continuous variables between the two groups. The Kruskal Wallis test was used to compare continuous variables between the three groups. Bonferroni correction was applied if there was a significant difference between groups and determine within-group differences. The Friedman test was used to compare repeated values of Fibulin 1, 2, and NT-proBNP levels to analyse temporal changes at the admission, discharge, and first month. Spearman’s test was used for correlation analyses. Logistic regression analysis was performed to determine whether Fibulins have any effect on short-term adverse events. SPSS Statistics for Windows, Version 23.0. (IBM Corp, Armonk, NY, USA.) was used for the statistical analyses.

## 3. Results

Seventy-five patients were included in the study between November 2021 and November 2022. 26 of the patients were in the reduced LVEF group, 24 of the patients were in the non-reduced LVEF group, and 25 of the patients were in the control group. Table 1 presents the demographic and laboratory data of the groups. Serum Fibulin 2 levels were similar between the groups. Serum Fibulin 1 and NT-proBNP levels were significantly higher in the patient group than in the control group (Table 1).

**Table 1.** **Demographics characteristics, medications, and laboratory data of the study population**.

	HFrEF (n: 26)	HFnrEF (n: 24)	Control Group (n: 25)	*p* value
Age, years	78 (68–83)	67 (59–74)	57 (51–67)	<0.001
Gender (male), n (%)	15 (57.7)	10 (41.7)	15 (60)	0.376
Hypertension, n (%)	17 (65.4)	21 (87.5)	21 (84)	0.118
Diabetes, n (%)	17 (65.4)	12 (50)	12 (48)	0.394
Ischemic heart disease, n (%)	26 (73.1)	24 (45.8)	13 (52)	0.121
Smoking, n (%)	4 (15.4)	1 (4.2)	13 (52)	<0.001
Beta blocker, n (%)	26 (100)	21 (87)	12 (48)	NA
ACEI/ARB/ARNI	23 (88.5)	15 (62.5)	20 (80)	
Loop diuretics	26 (100)	24 (100)	0	
MRA	22 (84.6)	7 (29.2)	0	
SGLT2 inhibitor	4 (15.4)	4 (16.6)	1 (4)	
NYHA Class I	0	0	25 (100)	NA
NYHA Class II	4 (15.4)	2 (8.3)	0	
NYHA Class III	20 (76.9)	19 (79.2)	0	
NYHA Class IV	2 (7.7)	3 (12.5)	0	
LVEF %	26 (21–30)	54 (50–59)	60 (60–65)	<0.001
Hemoglobin, g/dL	12.2 (10–14)	11.8 (9.3–13)	14.2 (12.8–15.5)	<0.001
White blood cells, ×10^3^ µL	7.2 (6.2–8.4)	8.3 (7.0–9.3)	6.8 (6.0–8.6)	0.250
Thrombocyte count, ×10^3^ µL	196 (169–259)	209 (170–264)	253 (240–390)	0.014
BUN, mg/dL	32 (22–48)	25 (21–36)	15 (12–19)	<0.001
Creatinine, g/dL	1.17 (0.82–1.51)	0.94 (0.81–1.18)	0.94 (0.81–1.05)	0.094
Sodium, mEq/L	138 (136–141)	139 (137–141)	140 (139–142)	0.085
Potassium, mEq/L	4.0 (3.6–4.6)	4.3 (3.9–4.7)	4.4 (4.0–4.6)	0.129
Total cholesterol, mg/dL	144 (99–178)	133 (109–162)	196 (160–224)	<0.001
LDL cholesterol, mg/dL	77 (57–105)	73 (55–92)	114 (84–149)	0.006
HDL cholesterol, mg/dL	29 (26–39)	38 (30–48)	41 (34–49)	0.019
Triglyceride, mg/dL	82 (67–114)	89 (75–117)	169 (124–198)	<0.001
NT-proBNP, pg/mL	6723 (4343–15,873)	3403 (1573–5691)	49 (39–128)	<0.001
Fibulin 1, ng/mL	130.4 (100.9–206.0)	142.9 (95.6–190.5)	47.2 (13.4–79.6)	<0.001
Fibulin 2, ng/mL	12.3 (8.4–21.1)	10.6 (7.3–15.0)	9.5 (5.6–12.7)	0.274

ACEI, angiotensin-converting enzyme inhibitor; ARB, angiotensin receptor blocker; ARNI, angiotensin receptor/neprilysin inhibitör; BUN, blood urea nitrogen; HFrEF, heart failure with reduced ejection fraction; HFnrEF, heart failure with non-reduced ejection fraction; LVEF, left ventricle ejection fraction; MRA, mineralocorticoid receptor antagonist; NYHA, New York Heart Association; LDL, low-density lipoprotein; HDL, high-density lipoprotein; NT-proBNP, N-terminal pro-B-type natriuretic peptide; SGLT2, sodium-glucose transport protein 2.

Temporal changes in serum Fibulin 1, Fibulin 2, and NT-proBNP levels for the whole patient group are presented in Table 2. Although serum Fibulin 2 levels were similar at repeated measurements, serum Fibulin 1 and NT-proBNP levels significantly decreased at discharge and sustained similar levels after one month compared with admission. These findings were consistent generally in reduced and non-reduced LVEF groups (Table 2).

**Table 2.** **Laboratory measurements ​​of patients at hospitalization, discharge and in first month**.

		Hospitalization	Discharge	First month	*p* value
Whole patient group	NT-proBNP, pg/mL	5052 (2691–8021)	3528 (1826–5139)	3007 (1636–4839)	0.001
	Fibulin-1, ng/mL	135.7 (100.9–206.0)	45.2 (15.7–128.5)	50.9 (27.8–77.6)	<0.001
	Fibulin-2, ng/mL	11.9 (7.6–18.2)	16.0 (8.7–26.1)	15.0 (10.0–24.7)	0.224
Reduced LVEF group	NT-proBNP, pg/mL	6723 (4343–15,873)	4476 (2743–11,358)	3685 (2144–15,765)	0.011
	Fibulin-1, ng/mL	130.4 (100.9–206.1)	79.1 (21.9–156.2)	51.6 (37.2–94.2)	0.001
	Fibulin-2, ng/mL	12.3 (8.4–21.1)	16.3 (9.4–30.3)	16.6 (12.8–30.4)	0.102
Non-reduced LVEF group	NT-proBNP, pg/mL	3403 (1573–5691)	2285 (800–3937)	2089 (1453–3260)	0.097
	Fibulin-1, ng/mL	142.9 (96.5–190.5)	31.7 (10.4–94.3)	48.8 (27.6–70.5)	<0.001
	Fibulin-2, ng/mL	10.6 (7.2–17.0)	10.9 (6.2–19.7)	12.8 (9.4–19.8)	0.152

LVEF, left ventricle ejection fraction; NT-proBNP, N-terminal pro-B-type natriuretic peptide.

**Fig. 1.**
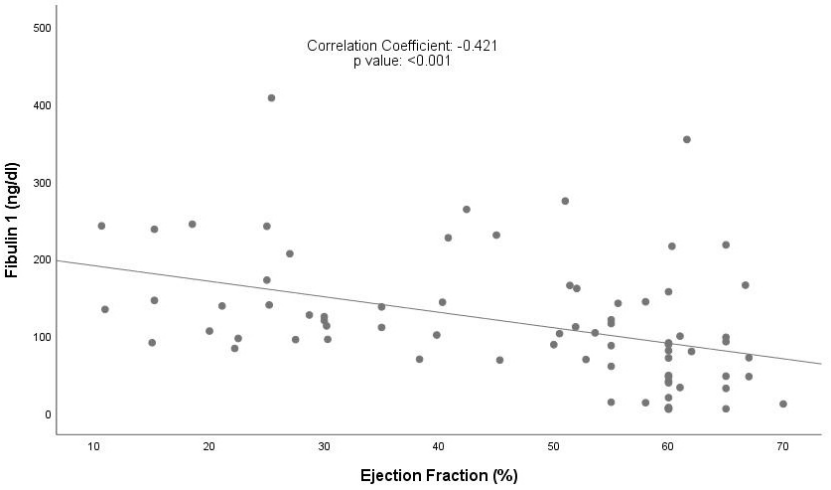
**Correlation between Fibulin 1 levels and LVEF**. LVEF, left ventricle ejection fraction.

**Fig. 2.**
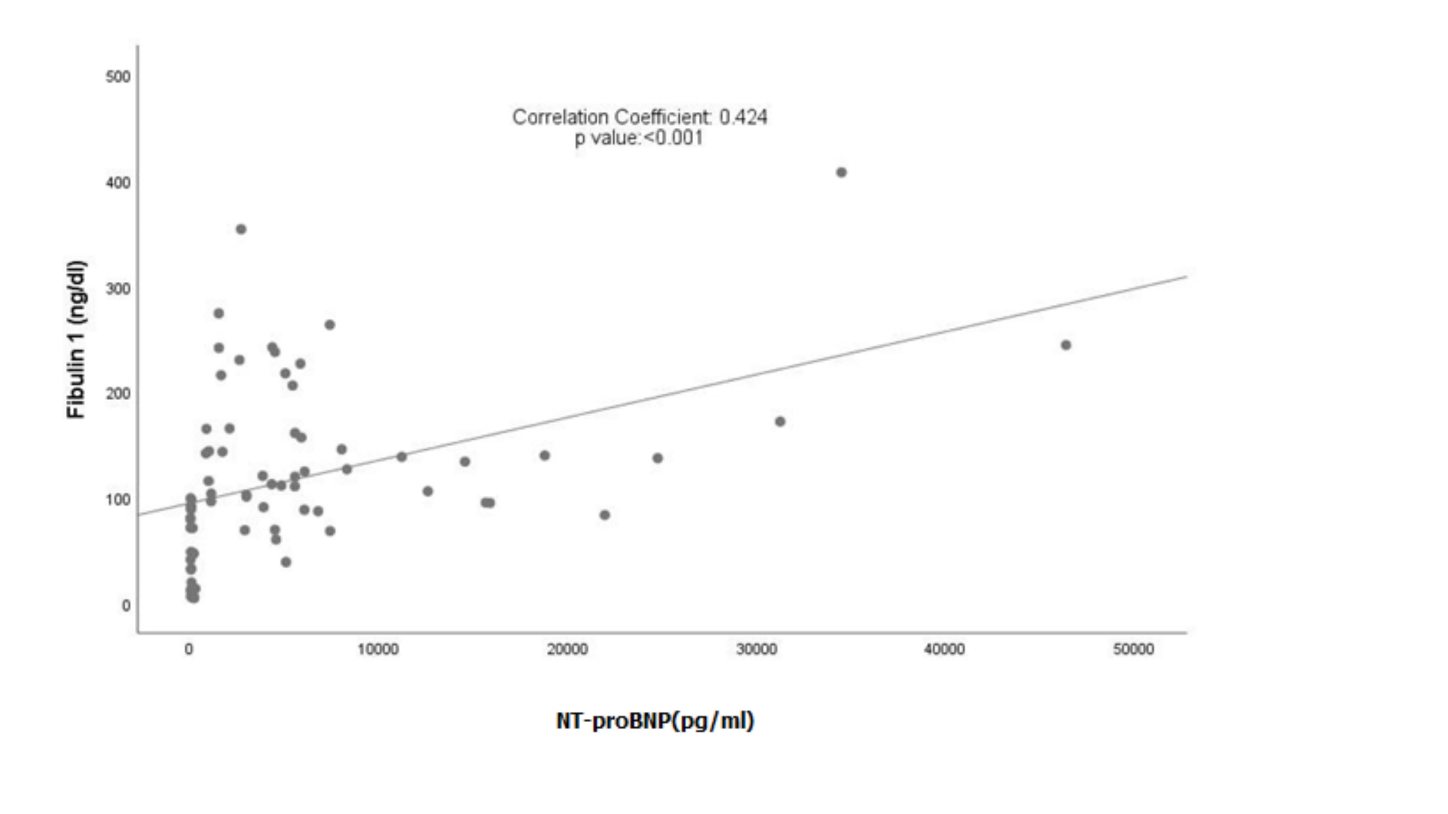
**Correlation between Fibulin 1 levels and NT-proBNP levels**. NT-proBNP, N-terminal pro-B-type natriuretic peptide.

There was a significant positive correlation between Fibulin 1 and NT-proBNP levels and a significant negative correlation between Fibulin 1 levels and LVEF (Figs. 1,2). However, when the same correlation analysis was performed for specific HF groups, the significance disappeared for both reduced and non-reduced LVEF groups (Table 3). Fibulin 2 levels were not correlated with LVEF or NT-proBNP (correlation coefficient –0.161 and 0.162, respectively).

**Table 3.** **Correlation between Fibulin 1 and NT-proBNP and LVEF in preserved and non-preserved LVEF groups**.

		Correlation coefficient	*p* value
Reduced LVEF group	NT-proBNP	0.173	0.397
	LVEF	–0.154	0.454
Non-reduced LVEF group	NT-proBNP	–0.287	0.174
	LVEF	0.095	0.659

LVEF, left ventricular ejection fraction; NT-proBNP, N-terminal pro-B-type natriuretic peptide.

Predefined adverse study outcomes were observed in 25 patients. Five individuals in the patient group died. Logistic regression analyses demonstrated that age and admission blood urine nitrogen levels were independent predictors of adverse outcomes in our study population. NT-proBNP level, Fibulin 1 level, and LVEF were not associated with study outcomes (Table 4).

**Table 4.** **Variables predicting study endpoints**.

	Exp(B)	95% CI		*p* value
		Lower	Upper	
Age	1.105	1.030	1.185	0.006
Gender	1.275	0.294	5.529	0.746
Hypertension	0.957	0.160	5.707	0.961
Diabetes	4.549	0.882	23.450	0.070
Fibulin-1 on hospitalization	1.007	0.998	1.017	0.143
BUN on hospitalization	1.092	1.014	1.175	0.020
NT-proBNP on hospitalization	1.000	1.000	1.000	0.845
LVEF on hospitalization	1.016	0.964	1.072	0.547

BUN, blood urea nitrogen; LVEF, left ventricular ejection fraction; NT-proBNP, N-terminal pro-B-type natriuretic peptide; Exp (B), or odds ratio, is the predicted change in odds for a unit increase in the predictor.

## 4. Discussion

This study evaluated Fibulin 1 and 2 levels in a population of symptomatic hospitalized patients with HF and patients with risk factors for HF but no prior symptoms. The results provided remarkable findings on Fibulin 1. Fibulin 1 levels were significantly higher in symptomatic HF patients compared to control patients. Admission Fibulin 1 levels decreased significantly at discharge and in the first month. These findings were similar for NT-proBNP levels in the study population. However, Fibulin 2 levels were similar between the study groups, and there was no significant temporal change in Fibulin 2 levels in symptomatic patients with HF.

HF is one of the most important public health problems, and its prevalence is increasing [10, 11, 12]. Myocardial fibrosis, whether under reduced or preserved LVEF conditions, has a causal link with the severity and the prognosis of HF [13]. Exploring the mechanism of the fibrotic process in the development of HF could help intervene in the progressive nature of the disease. Fibulin 1 is an ECM glycoprotein present in elastic fibers and the basement membrane. As ECM is the main object of fibrosis during remodelling, the particular role of Fibulin 1 in cardiac and vascular remodeling is under active research. Patients with diabetes are at risk of cardiac and vascular fibrosis. Cangemi *et al*. [14] evaluated the role of Fibulin 1 in vascular remodeling in patients with diabetes. They reported that plasma Fibulin 1 levels were higher in these patients. Fibulin 1 concentration was higher in diabetic artery extracts and increased Fibulin 1 immunostaining was apparent around the external elastic lamina of diabetic arteries. This study suggested that Fibulin 1 is actively present in vascular remodeling. The association between HF and Fibulin 1 has also been investigated in several clinical trials. Holmager *et al*. [15] evaluated Fibulin 1 levels in diabetic HF patients. They found that Fibulin 1 levels were elevated in patients with HF and impaired glucose metabolism. NT-proBNP is one of the most important prognostic and diagnostic markers of HF. Fibulin 1 was found to be associated with NT-proBNP levels in patients with aortic stenosis, HF, and a population composed of African individuals [15, 16, 17]. Our findings support those from previous studies. We found a positive correlation between Fibulin 1 and pro-B-type natriuretic peptide (Pro-BNP) and a negative correlation between Fibulin 1 and LVEF. In addition to the current literature, our study demonstrated that Fibulin 1 levels significantly decreased with HF treatment. We also observed a similar decrease in NT-proBNP levels with treatment, as expected. HF treatment decreases preload and afterload. Additionally, renin-angiotensin-aldosterone blockage and sympathetic nervous system blockage affect fibrotic processes. Both hemodynamic changes and pharmacological effects of medications could have been the cause of the changes in Fibulin 1 levels in our study. Oxlund *et al*. [18] demonstrated that spironolactone treatment reduced Fibulin 1 levels in patients with diabetic-resistant hypertension. Metformin similarly reduced Fibulin 1 levels in patients with diabetes [19]. However, there is also a contradictory result in the literature on Fibulin 1 in patients with HF. Eleuteri *et al*. [20] found that Fibulin 4 but not Fibulin 1 levels were higher in patients with HF than in controls. Our results demonstrated that neither Fibulin 1 nor NT-proBNP was associated with short term predefined study outcomes. The prognostic value of NT-proBNP in patients with HF is a well documented reality [21]. Our study was not designed for outcome analysis and was therefore underpowered for this purpose. This finding regarding NT-proBNP is a type II error related to the study design. Therefore, the findings between Fibulin 1 and study outcomes should be interpreted in this context. Dahl *et al*. [22] evaluated the prognostic role of Fibulin 1 in patients who underwent aortic valve replacement due to severe aortic stenosis. They found that patients who were in the highest serum Fibulin 1 tertile had significantly higher cardiac mortality during the median four year follow up. Although our findings cannot be interpreted in a prognostic manner, demonstrating how Fibulin 1 levels differ in different HF stages, how Fibulin 1 levels change in hospitalized patients with HF, and how Fibulin 1 levels are associated with NT-proBNP make our study valuable.

The results for Fibulin 2 did not exhibit similar changes to those for Fibulin 1 and NT-proBNP in our study. However, there is information in the literature showing that Fibulin 2 exhibits abnormal tissue expression and increased serum levels in some heart diseases associated with fibrosis. Fibulin 2 levels are significantly increased in serum and are abnormally expressed in the myocardium of hypertrophic cardiomyopathy patients [8]. Some experimental models have also supported the role of Fibulin 2 in cardiac remodeling [23]. The responses of Fibulin 1 and Fibulin 2 can differ under identical hemodynamic or pharmacological conditions. For example, although acute liver injury significantly increased Fibulin 1 expression; specific mRNA levels and immunohistochemical expression of Fibulin 2 remained unchanged throughout tissue injury and repair in an experimental study [24]. We think that our results are similar to the findings in this study.

Our study has important limitations that should be mentioned. First, the sample size was small. This could cause a potential type II error in Fibulin 2 results between study groups. Additionally, the small sample size hindered the division of patients into three groups, so we gathered patients with HF with mildly reduced LVEF and HF with preserved LVEF. Second, although we recorded predefined patient outcomes, our study was not designed to evaluate the relationship between fibulins and long-term cardiovascular outcomes. Therefore, our study cannot comment on the prognostic role of Fibulin 1 and 2 in patients with HF. However, repeated measurements of Fibulin 1 and 2 have provided important insights into the temporal changes in Fibulin 1 and 2 in HF patients. Additionally, the association between Fibulin 1 and NT-proBNP and LVEF could be a hypothesis generating finding for further studies.

## 5. Conclusions

Our study demonstrated that serum Fibulin 1 levels differ among different HF stages and have similar temporal changes as observed for NT-proBNP levels. A similar association was not observed for Fibulin 2 in our study. Our findings exhibited hopeful results about the diagnostic and prognostic use of Fibulin 1 in HF. Fibulin 1 could become a biomarker that could be used in daily clinical practice if further large scale studies support our findings.

## Availability of Data and Materials

The datasets used and/or analyzed during the current study are available from the corresponding author on reasonable request.
